# A Highly Sensitive Fluorescent Sensing Platform Utilizing Lanthanide Metal–Organic Frameworks for Total Antioxidant Detection

**DOI:** 10.3390/s26185711

**Published:** 2026-09-09

**Authors:** Wanyang Zhou, Yuanqiao He, Shangqing Zhang, Yafei Chen, Haiyan Li, Mingli Chen

**Affiliations:** 1 Department of Chemistry, College of Sciences, Northeastern University, Shenyang 110819, China; 2 College of Chemistry and Molecular Engineering, Northeast Agricultural University, Harbin 150030, China

**Keywords:** lanthanide MOFs, fluorescence, sensing, TAC

## Abstract

Total antioxidant capacity (TAC) reflects the collective ability of biological systems to counteract oxidative stress and is therefore an important indicator in redox biology, nutritional evaluation, and clinical assessment. Conventional TAC assays often suffer from insufficient sensitivity, pronounced matrix interference, and limited reliability in complex biological samples. Herein, we developed a redox-active lanthanide metal–organic framework (CeMOF@Tb) through mild aqueous-phase synthesis followed by post-synthetic incorporation of Tb^3+^ ions, serving as a luminescent probe for sensitive and reliable TAC analysis. The reversible Ce^4+^/Ce^3+^ redox couple serves as the antioxidant-responsive recognition unit, while Tb^3+^ provides a characteristic green luminescence output. The antioxidant-mediated reduction of Ce^4+^ to Ce^3+^ regulates the energy transfer process, thereby producing a concentration-dependent enhancement in Tb^3+^ emission and reduction in Ce^3+^ fluorescence. The sensing platform also exhibits high selectivity and strong resistance to interference from common coexisting species, supporting reliable TAC determination in complex biological matrices. Furthermore, the probe was applied to human serum samples, yielding recoveries of 91.6–122.6%. This study establishes an integrated redox-to-luminescence transduction strategy for TAC analysis and provides a versatile design framework for developing Ln-MOF-based probes for clinical biochemical applications.

## 1. Introduction

Antioxidants comprise a diverse group of endogenous and exogenous bioactive substances that protect biological systems against oxidative damage. Their protective effects are mainly achieved through the direct scavenging of reactive oxygen and nitrogen species (ROS/RNS), interruption of oxidative chain reactions, and regulation of antioxidant enzymes and redox-active metabolites [1,2,3]. Total antioxidant capacity (TAC) represents the combined ability of all antioxidant components in a biological system to counteract oxidative stress [4,5]. Under pathological conditions, excessive ROS production disrupts the balance between oxidants and antioxidants, leading to sustained oxidative stress, which can induce membrane lipid peroxidation, protein oxidation and aggregation, and oxidative damage to both nuclear and mitochondrial DNA [6,7,8]. The resulting molecular and cellular functional disorders are closely associated with aging and the development of cardiovascular diseases, neurodegenerative disorders, and various cancers [9]. Consequently, accurate and reliable evaluation of TAC is essential to advance fundamental insights into redox biology, facilitate the screening of potent natural and synthetic antioxidants, and support the assessment of bioavailability for bioactive compounds in nutraceuticals, functional foods, and pharmaceutical formulations. Because TAC is an operational and assay-dependent parameter rather than the concentration of a single antioxidant, it is commonly quantified relative to a reference antioxidant. Ascorbic acid (AA) is widely used as a reference antioxidant and calibration standard in antioxidant-capacity assays, with the results commonly expressed as AA equivalents [10,11,12]. Therefore, AA was selected as the model antioxidant and calibration standard for evaluating the TAC response of the proposed platform, and the results were expressed as AA-equivalent TAC. This calibration does not imply that all antioxidant species exhibit identical molar responses toward the Ce^4+^/Ce^3+^-based sensing system.

To date, a wide range of strategies has been developed for TAC assessment, including colorimetry [13], fluorescence [14], electrochemical [15], and surface-enhanced Raman scattering (SERS) approaches [16], which can quantify TAC by converting the overall reducing and radical-scavenging activity of antioxidants into measurable optical or electrochemical signals, enabling applications in clinical diagnostics and food and pharmaceutical analysis [17,18]. Among these techniques, colorimetry assays are simple, cost-effective, and suitable for high-throughput analysis, but the sensitivity and accuracy are often compromised by colored sample matrices. Electrochemical methods provide rapid response and convenient instrumentation, yet they remain susceptible to electrode fouling and interference from coexisting electroactive species. SERS sensing platforms offer high sensitivity and molecular fingerprinting capability, although their practical application is limited by substrate variability and signal heterogeneity. Fluorescence sensing has emerged as a particularly attractive strategy for TAC determination because it offers high sensitivity, a broad linear range, rapid response, and operational simplicity [19]. However, conventional fluorescence dyes are often affected by photobleaching, variations in probe concentration and excitation intensity, and background fluorescence from biological samples, which compromise analytical accuracy and limit their use in complex physiological environments [20]. Accordingly, there is a need for TAC sensing platforms that combine high sensitivity and selectivity with stable signals and strong resistance to matrix interference.

Metal–organic frameworks (MOFs) are crystalline porous materials assembled from metal ions or clusters and organic ligands, offering high surface areas, tunable pore structures, and readily modifiable chemical environments [21,22]. Lanthanide ions (Ln^3+^) exhibit sharp emission bands, large Stokes shifts, long luminescence lifetimes, and good photostability, making them well suited as optical signal units [23,24,25]. Ln-MOFs integrating the distinct luminescence characteristics of Ln^3+^ with the structural stability of MOFs have been widely applied in gas adsorption and separation [26], catalysis [27], environmental monitoring [28], biosensing [29], tissue imaging [30], and tumor treatment [31]. The luminescence of Ln-MOFs can be enhanced through several sensitization pathways. Organic ligands commonly act as antennae by absorbing excitation light and transferring the energy to Ln^3+^ centers, thereby compensating for the weak direct absorption of the *f*-*f* transitions [32]. In addition, sensitizer ions such as Ce^3+^ can be introduced to broaden the excitation range through their allowed 4*f*-5*d* transitions and subsequently transfer energy to activator ions such as Tb^3+^ [33]. By combining ligand-mediated and ion-mediated sensitization, Ln-MOFs can achieve tunable emission, providing multiple optical channels for biosensing and improving detection reliability in complex samples.

Although Ce/Tb-based redox-luminescence sensing systems have previously been reported [34], the previous study mainly focused on threshold-like detection of specific enzyme biomarkers, whereas the present work extends this sensing principle to the direct and continuous quantitative evaluation of AA-equivalent TAC. In this work, we developed a redox-responsive Ln-MOF probe (CeMOF@Tb) for TAC detection. Using pyromellitic acid (PMA) as the linker, a series of isostructural CeMOFs were prepared using different nominal Ce^4+^/Ce^3+^ precursor ratios through mild aqueous coordination assembly at room temperature. Tb^3+^ ions were then introduced by post-synthetic coordination with the unoccupied carboxyl sites of the CeMOF framework, yielding the CeMOF@Tb fluorescence probe. Ce^4+^ is reduced to Ce^3+^ in the presence of ascorbic acid, which promotes energy transfer from Ce^3+^ sensitizers to Tb^3+^ emitters, resulting in enhanced Tb^3+^ emission at 545 nm. This fluorescence response represents the overall reducing capacity of the sample and enables quantitative TAC analysis. The method was further applied to TAC determination in human serum, demonstrating its potential for reliable analysis in complex biological samples.

## 2. Experimental Section

### 2.1. Chemicals and Apparatus

Chemicals: Pyromellitic acid hydrate (PMA), anhydrous cerium(III) chloride (CeCl_3_), terbium(III) chloride hexahydrate (TbCl_3_·6H_2_O), sodium chloride (NaCl), potassium chloride (KCl), and lysozyme were obtained from Macklin Biochemical Technology Co., Ltd. (Shanghai, China). Magnesium chloride (MgCl_2_), zinc chloride (ZnCl_2_), calcium chloride (CaCl_2_), sodium bicarbonate (NaHCO_3_), and sodium sulfate (Na_2_SO_4_) were obtained from Damao Chemical Reagent Co., Ltd. (Tianjin, China). L-glutamic acid (Glu), L-lysine (Lys), glucose, urea, glutathione (GSH), iron(III) chloride hexahydrate (FeCl_3_·6H_2_O), 1,10-phenanthroline monohydrate (C_12_H_8_N_2_·H_2_O) and N-ethylmaleimide (NEM) were obtained from Aladdin Biochemical Technology Co., Ltd. (Shanghai, China). Ammonium cerium(IV) nitrate ((NH_4_)_2_Ce(NO_3_)_6_), tripotassium orthophosphate (K_3_PO_4_), potassium thiocyanate (KSCN), ascorbic acid (AA), L-phenylalanine (Phe), glycine (Gly), and L-serine (Ser) were obtained from Sinopharm Chemical Reagent Co., Ltd. (Shenyang, China). Formic acid (FA) and triethylamine (TEA) were obtained from Shenyang Damao Chemical Reagent Co., Ltd. (Shenyang, China). L-proline (Pro), lactoferrin, and alkaline phosphatase (ALP) were obtained from Shanghai Yuanye Biotechnology Co., Ltd. (Shanghai, China). Immunoglobulin (IgG) was obtained from Sigma-Aldrich Shanghai Trading Co., Ltd. (Shanghai, China). Anhydrous alcohol (EtOH) was obtained from Tianjin Fuyu Fine Chemical Co., Ltd. (Tianjin, China). All chemicals were of analytical grade and used without further purification and modification. Deionized water (DI) of 18 MΩ·cm was used throughout.

Apparatus: The size, morphology and EDS elemental mappings of CeMOF and CeMOF@Tb were observed on a ZEISS GeminiSEM 560 ultra-high-resolution FESEM (Carl Zeiss Microscopy GmbH, Jena, Germany) and a Talos F200X G2 transmission electron microscope (Thermo Fisher Scientific, Hillsboro, OR, USA) using an accelerating voltage of 200 kV. UV-vis absorption spectra were collected by using a UV-3600i Plus spectrophotometer (Shimadzu Corporation, Kyoto, Japan) with a 1.0 cm quartz cell. Fourier transform infrared (FT-IR) spectra were obtained by using a VERTEX 70 FT-IR spectrophotometer (Bruker Optics GmbH & Co. KG, Ettlingen, Germany) from 400 to 4000 cm^−1^. X-ray photoelectron spectra (XPS) were obtained on an AXIS SUPRA spectrometer (Kratos Analytical Ltd., Manchester, UK). X-ray diffraction (XRD) patterns were collected on SmartLab (Rigaku Corporation, Akishima, Japan). The fluorescence spectra were recorded with an RF-6000 fluorescence spectrophotometer (Shimadzu Corporation, Kyoto, Japan). Lifetime decay curves were measured using a FLS 1000 fluorescence spectrophotometer (Edinburgh Instruments Ltd., Livingston, UK).

### 2.2. The Preparation of CeMOF

CeMOF was prepared based on the previously reported method and with modifications [34]. In total, 1.2 mmol (NH_4_)_2_Ce(NO_3_)_6_ and 0.8 mmol CeCl_3_ were dissolved in 8 mL, 5.25 mol/L formic acid (FA) aqueous solution. Meanwhile, 2 mmol of pyromellitic acid (PMA) was dispersed into a 12 mL FA (5.25 mol/L) solution containing 400 μL triethylamine (TEA). Subsequently, the two solutions were uniformly mixed and stirred at room temperature for 2 h to form a pale-yellow product. Next, the product was obtained by centrifugation (8000 rpm, 8 min), washed 3 times with ultrapure water and anhydrous ethanol, then soaked in ethanol for 48 h, with the ethanol being replaced every 12 h. The collected yellow product was dried in an oven at 60 °C overnight, yielding CeMOF prepared using a nominal n(Ce^4+^)/n(Ce^3+^) precursor molar ratio of 6:4.

The synthesis processes for the other proportions are the same as above, except that the amounts of (NH_4_)_2_Ce(NO_3_)_6_ and CeCl_3_ are different.

### 2.3. The Preparation of CeMOF@Tb

CeMOF samples (100 mg) prepared using different nominal Ce^4+^/Ce^3+^ precursor molar ratios were dispersed in 10 mL of TbCl_3_·6H_2_O aqueous solution (0.1 mol/L) and stirred at 60 °C for 24 h. Then, the product was obtained by centrifugation (10,000 rpm, 5 min), and washed 3 times with water. The collected product was dried in an oven at 60 °C overnight.

### 2.4. Optimization of Experimental Parameters for AA Detection

The effects of solution pH and incubation time on the fluorescence response of the CeMOF@Tb probe were investigated sequentially. For optimization of solution pH, 50 μL of the CeMOF@Tb aqueous dispersion (500 μg/mL) and 50 μL of AA solution were sequentially added to a 0.5 mL microcentrifuge tube. The final AA concentration was fixed at 100 μM, and the total reaction volume was adjusted to 250 μL using 10 mM HEPES solutions with pH values ranging from 4.0 to 8.0. A corresponding blank was prepared at each pH by replacing the AA solution with an equal volume of ultrapure water. After thorough mixing, the reaction mixtures were incubated at room temperature for 120 s before fluorescence measurement. The fluorescence enhancement at each pH was calculated as ΔF_545_ = F_545_, AA − F_545_, where F_545_, AA and F_545_ represent the fluorescence intensities at 545 nm in the presence and absence of AA, respectively.

After selection of the appropriate pH, the fluorescence response as a function of incubation time was examined using the same reaction composition at pH 6.0. Immediately after the addition of AA, the fluorescence intensity at 545 nm was recorded at 0, 10, 20, 30, 60, 120, 180, 240, 300, 360, 420, 480, 540, and 600 s without subtraction of the blank signal. All fluorescence measurements were performed at an excitation wavelength of 280 nm, with both excitation and emission slit widths set to 10 nm. Following optimization, pH 6.0 and an incubation time of 120 s were selected for subsequent fluorescence measurements.

### 2.5. Fluorescence Determination of AA with the CeMOF@Tb Probe

For fluorescence determination of AA, 50 μL of the CeMOF@Tb aqueous dispersion (500 μg/mL) was transferred into a 0.5 mL microcentrifuge tube, followed by the addition of 50 μL of an AA standard solution at the required concentration. Subsequently, 150 μL of 10 mM HEPES solution at pH 6.0 was added to obtain a final reaction volume of 250 μL. The resulting mixture was thoroughly mixed and incubated at room temperature for 120 s.

Fluorescence spectra were recorded using an excitation wavelength of 280 nm, with both excitation and emission slit widths set to 10 nm. The fluorescence intensity at 545 nm was used as the analytical signal. A reagent blank was prepared under identical conditions by replacing the AA standard solution with 50 μL of ultrapure water. The fluorescence response was expressed as F/F_0_, where F and F_0_ represent the fluorescence intensities at 545 nm in the presence and absence of AA, respectively. AA standard solutions giving final concentrations within the range of 5–200 μM were analyzed, and the calibration curve was constructed by plotting F/F_0_ against the final AA concentration. All measurements were performed independently in triplicate.

For the selectivity experiment, AA and each potential interfering substance were individually introduced into separate CeMOF@Tb sensing mixtures and analyzed according to the procedure described above. For evaluation of interference resistance, AA and each potential interfering substance were simultaneously introduced into the sensing system. In experiments involving GSH, the GSH solution was treated with NEM before being added to the sensing mixture to block the free thiol group. NEM was not added to samples containing the other potential interfering substances.

### 2.6. Analytical Procedure for Determining AA-Equivalent Antioxidant Capacity in Human Serum Samples

The applicability of the fluorescence sensing strategy to biological samples was evaluated by determining AA-equivalent antioxidant capacity in human serum. Blood samples were obtained from the School Hospital of Northeastern University, and all experiments were conducted in accordance with relevant institutional guidelines and regulations. Serum was separated by centrifugation before detection. For each measurement, 50 μL of the serum sample, 50 μL of AA standard solution at the desired concentration, and 50 μL of CeMOF@Tb dispersion (500 μg/mL) were added to a 0.5 mL microcentrifuge tube. The final volume was adjusted to 250 μL with 10 mM HEPES solution at pH 6.0, followed by thorough mixing and incubation for 120 s at room temperature. The serum samples were used without prior dilution; the introduction of 50 μL of serum into the final 250 μL assay mixture resulted in a fivefold dilution. The accuracy and reliability of the method were evaluated using standard addition recovery experiments. All recovery experiments were performed independently in triplicate (n = 3). The AA-spiking procedure was used to evaluate analytical recovery and potential matrix effects, whereas samples with an AA addition level of 0 μM were directly analyzed to determine their endogenous AA-equivalent antioxidant capacity. For independent validation, the same unspiked serum samples were analyzed using the Fe^3+^/1,10-phenanthroline UV-vis reference assay according to the procedure described in Text S1 of the Supporting Information. The absorbance at 510 nm was converted to an AA-equivalent concentration using the corresponding calibration curve.

All determinations were performed in triplicate and are reported as mean ± standard deviation (SD). Comparisons between the proposed CeMOF@Tb method and the UV-vis reference method were conducted using separate two-sided unpaired Welch’s *t*-tests in GraphPad Prism 10.1.2, with *p* < 0.05 considered statistically significant.

## 3. Results and Discussion

### 3.1. Preparations and Characterizations of CeMOF and CeMOF@Tb

**Scheme 1:**
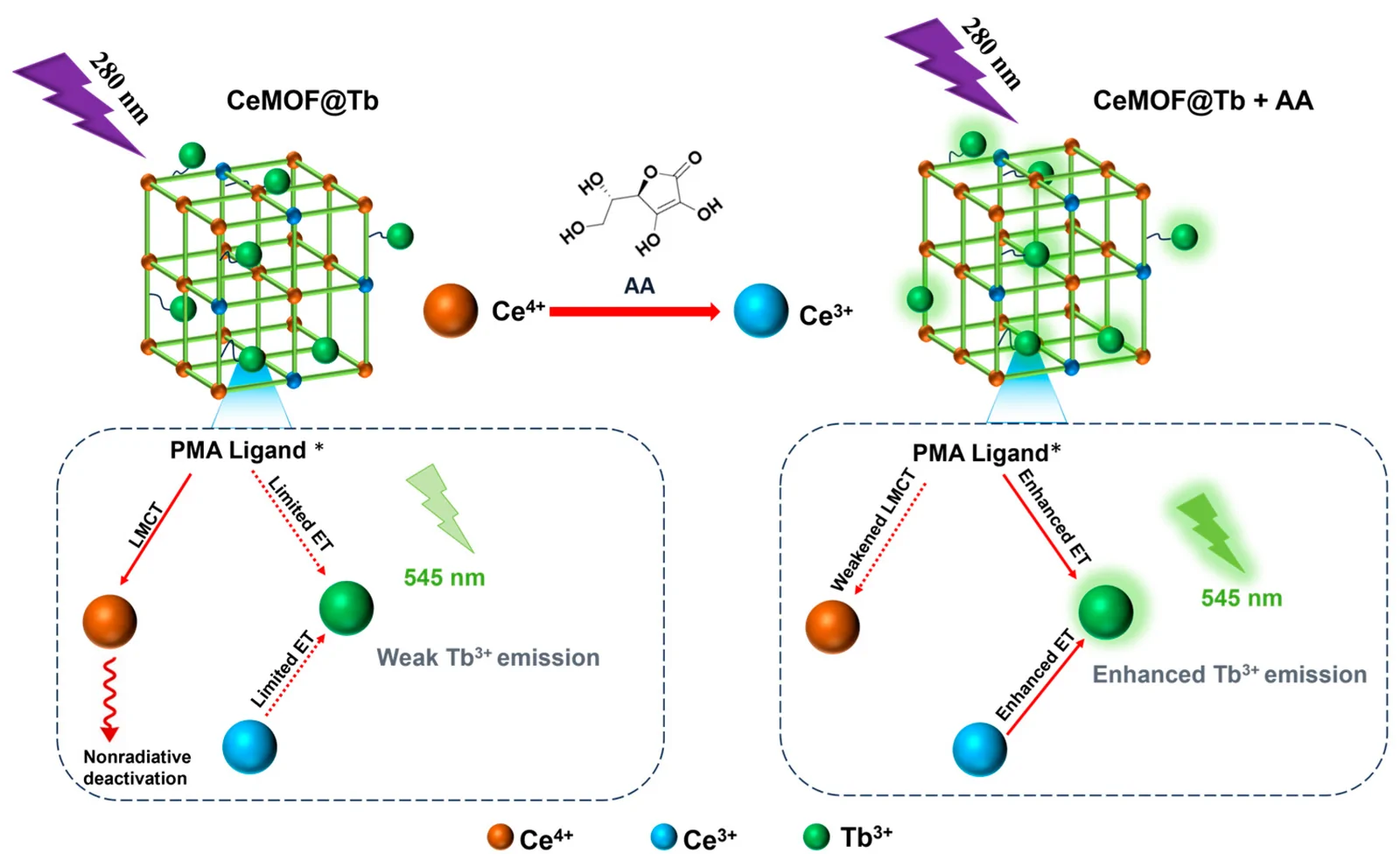
Schematic illustration of the proposed luminescence response mechanism of CeMOF@Tb toward AA. The asterisk denotes the excited state of the PMA ligand. LMCT and ET denote ligand-to-metal charge transfer and energy transfer, respectively. Solid and dashed red arrows represent effective and weak transfer pathways, respectively, whereas the red wavy arrow indicates nonradiative deactivation.

**Figure 1:**
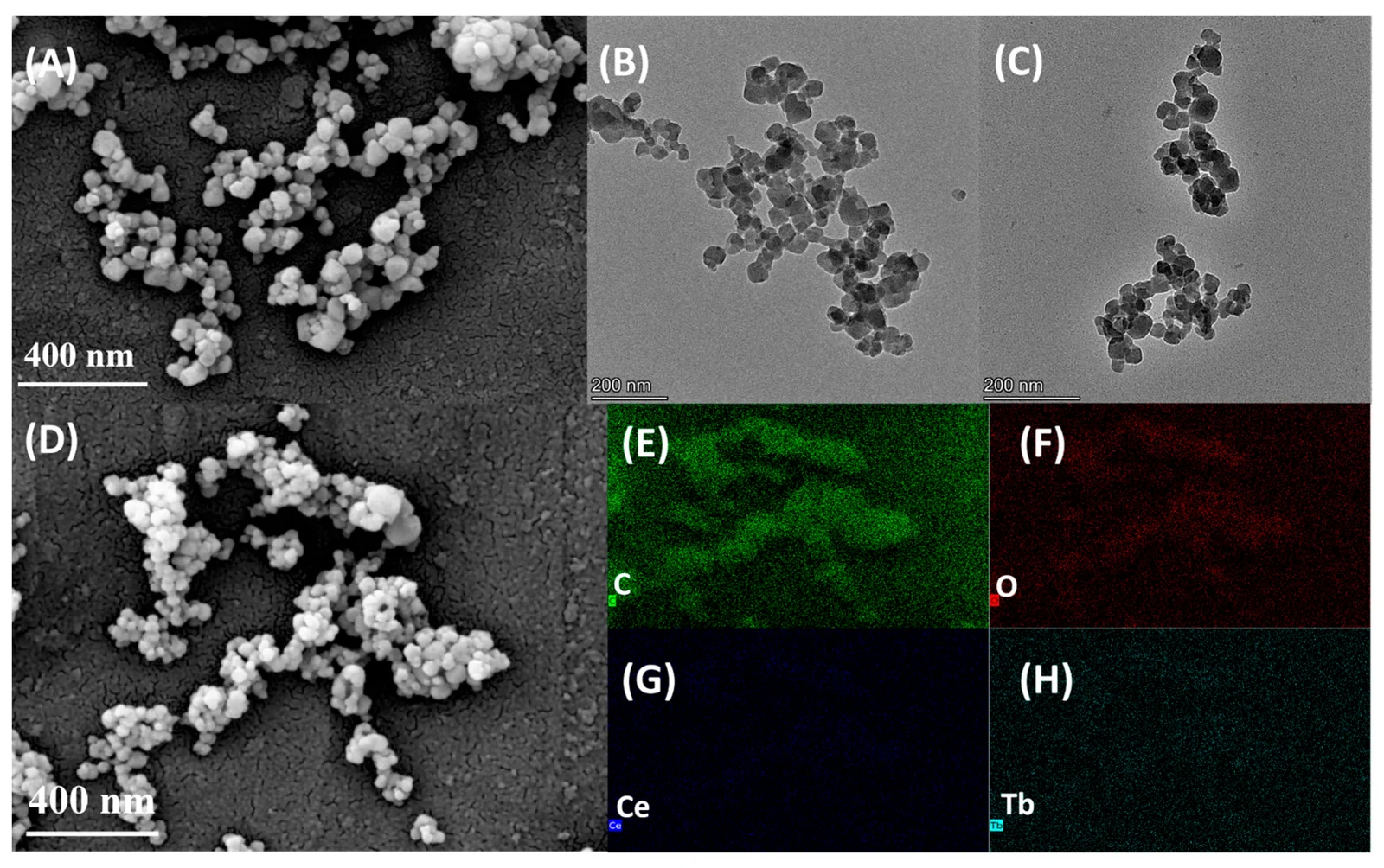
SEM images of CeMOF (**A**) and CeMOF@Tb (**D**). TEM images of CeMOF (**B**) and CeMOF@Tb (**C**). EDS elemental mapping images of C (**E**), O (**F**), Ce (**G**), and Tb (**H**) in CeMOF@Tb.

The proposed luminescence response mechanism of CeMOF@Tb toward AA is schematically illustrated in Scheme 1. In this study, CeMOFs were synthesized using different nominal Ce^4+^/Ce^3+^ precursor molar ratios through an aqueous room-temperature coordination self-assembly strategy, with PMA serving as the organic ligand and Ce^4+^/Ce^3+^ as the metal centers. Subsequently, Tb^3+^ ions were coordinated to the remaining uncoordinated carboxyl sites on the CeMOF framework through post-synthetic modification strategy, resulting in the CeMOF@Tb probe. The morphologies and sizes of synthesized CeMOFs were characterized by scanning electron microscopy (SEM) and transmission electron microscopy (TEM). SEM images showed that CeMOFs prepared using different nominal Ce^4+^/Ce^3+^ precursor molar ratios exhibited clear and highly uniform cubic morphologies (Figure 1A and Figure S1). After functionalization with Tb^3+^, no significant morphological changes were observed, as confirmed by comparison of TEM images (Figure 1B,C). Energy-dispersive X-ray spectroscopy (EDS) elemental mapping images clearly demonstrated the homogeneous distribution of C, O, Ce, and Tb on the CeMOF@Tb, demonstrating the successful and uniform anchoring of Tb^3+^ ions (Figure 1E–H).

**Figure 2:**
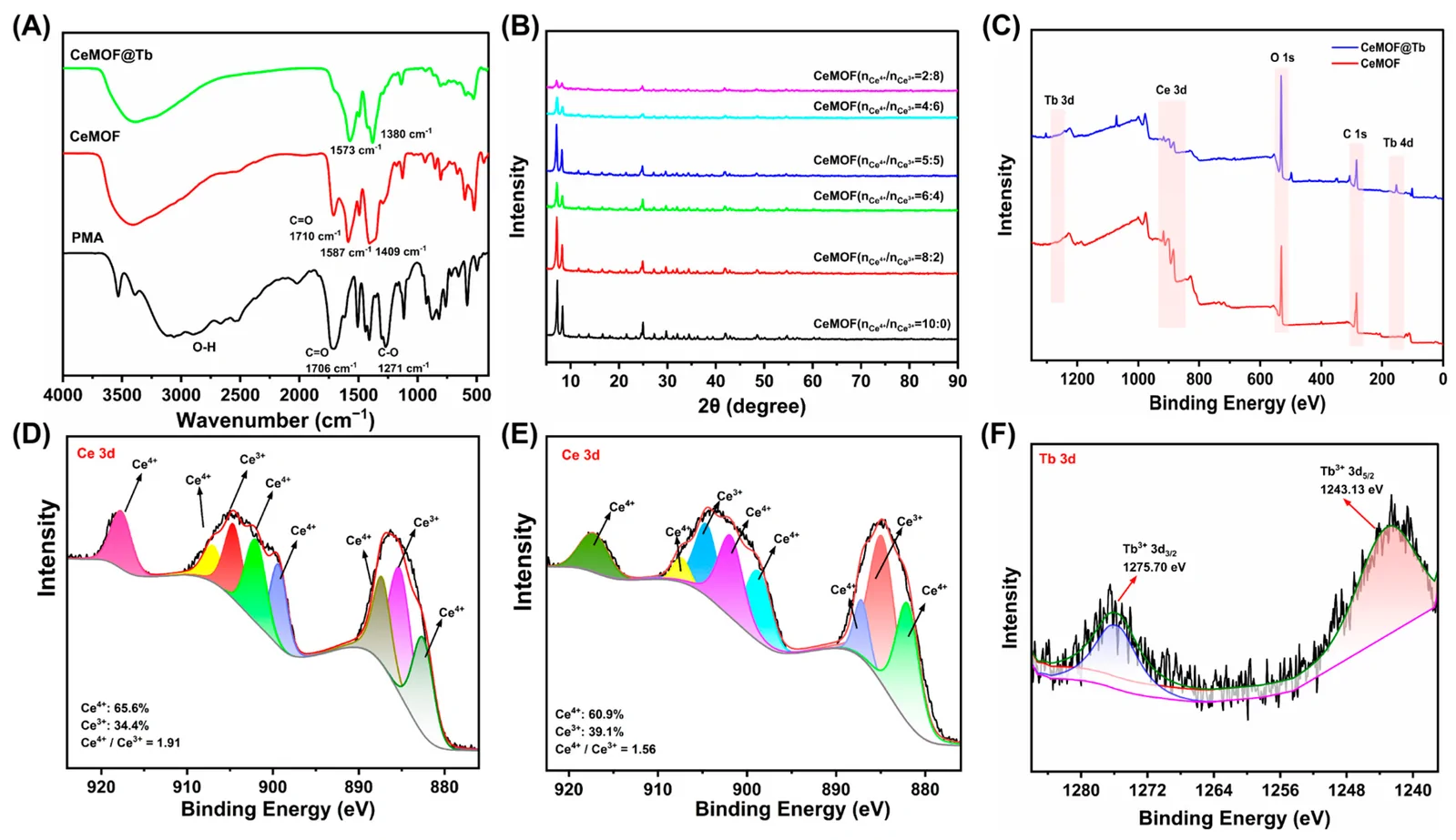
(**A**) FT-IR spectra of PMA, CeMOF, CeMOF@Tb. (**B**) X-ray diffraction (XRD) patterns of CeMOF prepared using different nominal Ce^4+^/Ce^3+^ precursor molar ratios. (**C**) XPS survey spectra of CeMOF and CeMOF@Tb. High-resolution Ce 3d spectra of (**D**) CeMOF and (**E**) CeMOF@Tb, and (**F**) high-resolution Tb 3d spectrum of CeMOF@Tb. In (**D**–**F**), the experimental spectra, overall fits, deconvoluted components, and fitted backgrounds are shown in black, red/green, multiple colors, and gray/magenta, respectively.

Fourier transform infrared (FTIR) spectroscopy was used to examine the coordination of PMA with Ce^4+^/Ce^3+^ and Tb^3+^. Compared with free PMA, the characteristic C=O stretching band of protonated carboxyl groups at 1706 cm^−1^ was shifted to 1710 cm^−1^, and markedly weakened in the CeMOF spectrum. Meanwhile, absorption bands at 1587 and 1409 cm^−1^ assigned to the asymmetric and symmetric stretching vibrations of coordinated carboxylate groups were observed [35]. A weak feature in the 3100–3000 cm^−1^ region may be attributed to the aromatic C-H stretching vibrations of the PMA ligand. This feature overlaps with the broad O-H stretching absorption associated with residual carboxylic acid groups and adsorbed water in the approximately 3500–2500 cm^−1^ region. After Tb^3+^ modification, the residual C=O stretching band of the uncoordinated carboxylic acid groups at 1710 cm^−1^ disappeared, while the asymmetric and symmetric COO^-^ stretching bands shifted from 1587 and 1409 cm^−1^ in CeMOF to 1573 and 1380 cm^−1^ in CeMOF@Tb, respectively. These changes indicate deprotonation of the residual carboxylic acid groups and coordination of Tb^3+^ through carboxylate oxygen atoms. The Δν value increased from 178 to 193 cm^−1^, which is consistent with a substantial contribution from monodentate coordination [36]. However, Δν alone cannot provide an unambiguous assignment when several nonequivalent carboxylate sites are present [37]. Considering that pyromellitate ligands can also adopt bridging modes in structurally characterized Tb^3+^ coordination polymers [38], minor chelating or bridging contributions cannot be excluded. Therefore, the FTIR results suggest predominantly monodentate coordination of Tb^3+^ at the residual carboxylate sites (Figure 2A). Powder X-ray diffraction (PXRD) was used to evaluate the crystallinity and structural stability of the materials. CeMOFs prepared using different nominal Ce^4+^/Ce^3+^ precursor molar ratios displayed sharp diffraction peaks at 7.2° and 8.3°, indicating good crystallinity. After Tb^3+^ modification, CeMOF@Tb retained the characteristic diffraction peaks of CeMOF, while the intensities decreased slightly, indicating that the introduction of Tb^3+^ reduced its crystallinity (Figure 2B and Figure S2). X-ray photoelectron spectroscopy (XPS) measurements were carried out to analyze the elemental composition and chemical valence states of CeMOF@Tb. The full XPS survey spectrum showed distinct characteristic peaks of C 1s, O 1s, Ce 3d, and Tb 3d, which is in good agreement with the EDS elemental analysis (Figure 2C). In the high-resolution Tb 3d spectrum, two characteristic peaks at 1243.13 and 1275.70 eV were assigned to Tb 3d_5/2_ and Tb 3d_3/2_, respectively (Figure 2F) [35]. The high-resolution Ce 3d spectrum can be deconvoluted into characteristic peaks of Ce^4+^ and Ce^3+^, confirming the coexistence of mixed-valence Ce species in the CeMOF@Tb, which provides the structural basis for its redox-responsive properties. Quantitative analysis of the fitted Ce 3d peak areas showed that the Ce^4+^ and Ce^3+^ surface fractions were 65.6% and 34.4% for CeMOF and 60.9% and 39.1% for CeMOF@Tb, respectively. The corresponding Ce^4+^/Ce^3+^ surface ratios were calculated to be 1.91 and 1.56 (Figure 2D,E). These XPS-derived values represent the surface-oxidation-state composition, whereas the 6:4 ratio refers to the nominal precursor molar ratio used during synthesis.

### 3.2. Optical Properties and Response Mechanism of CeMOF@Tb

**Figure 3:**
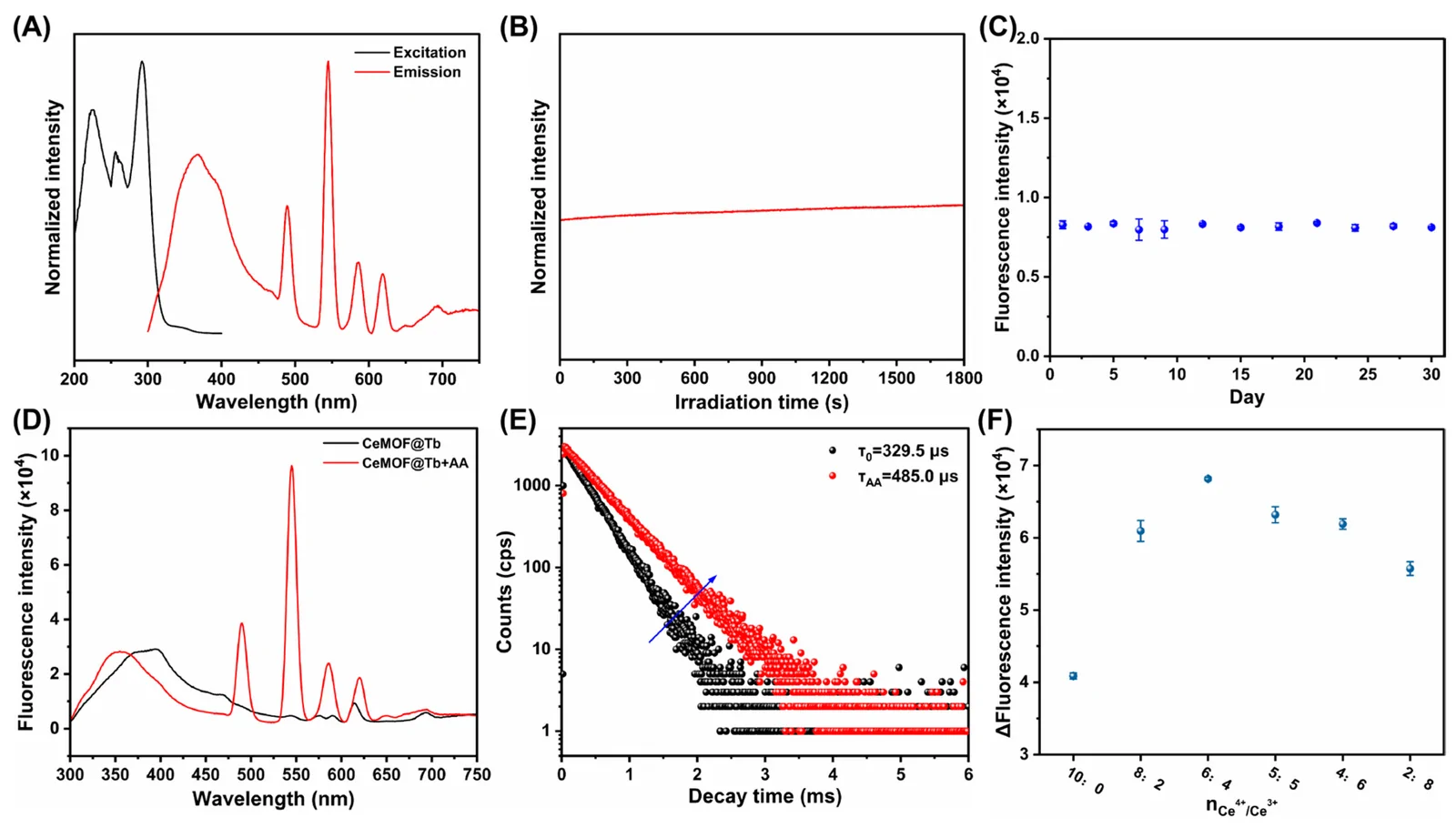
(**A**) Excitation (λ_em_ = 545 nm) and emission (λ_ex_ = 280 nm) spectra of CeMOF@Tb. (**B**) The photobleaching resistance of CeMOF@Tb under continuous light excitation for 30 min. (**C**) The interdaily stability of the fluorescence signal of CeMOF@Tb. (**D**) The feasibility of detecting AA using the CeMOF@Tb probe. (**E**) Lifetime decay curves of the Tb^3+^ emission of the CeMOF@Tb probe at 545 nm before (black dots) and after (red dots) addition of AA The blue arrow indicates the increase in the Tb^3+^ luminescence lifetime after AA addition. (**F**) Optimization of the molar-ratio of Ce^4+^/Ce^3+^ for the fluorescence sensing system.

The photoluminescence excitation and emission properties of CeMOF@Tb were examined to determine the optimal optical conditions. The excitation spectra showed two distinct bands centered at 256 and 290 nm under 545 nm emission, which were attributed to the π-π* transition of aromatic PMA ligand and a charge-transfer transition associated with Ce centers, respectively (Figure 3A). The emission spectra displayed the four characteristic Tb^3+^ transitions at 490, 545, 585, and 625 nm, assigned to the ^5^D_4_ → ^7^F_6_, ^5^D_4_ → ^7^F_5_, ^5^D_4_ → ^7^F_4_, and ^5^D_4_ → ^7^F_3_ transitions, respectively. Furthermore, a distinct emission band near 350 nm was also observed and attributed to the allowed 5d → 4f transition of Ce^3+^. The emission band centered at 545 nm exhibited the highest intensity among all Tb^3+^ emission bands and was thus selected as the quantitative signal (F_545_). The photostability of the CeMOF@Tb probe was evaluated under continuous UV irradiation. The fluorescence signal remained nearly unchanged over 30 min, indicating that the probe exhibited excellent resistance to photobleaching (Figure 3B). Meanwhile, the daytime stability results showed that the fluorescence signal of the probe exhibited only negligible fluctuations during 30 days of storage, demonstrating excellent long-term storage stability (Figure 3C).

Subsequently, the feasibility of the CeMOF@Tb probe for evaluating TAC was investigated using AA as a model antioxidant and calibration standard. The proposed luminescence response involves an AA-regulated redox process coupled with Ce^3+^-sensitized Tb^3+^ emission. Owing to the insufficient sensitization by the PMA ligand, CeMOF@Tb initially exhibited weak Tb^3+^ emission at 545 nm. After AA treatment, high-resolution Ce 3d XPS analysis showed that the Ce^4+^ fraction decreased from 60.9% to 57.2%, whereas the Ce^3+^ fraction increased from 39.1% to 42.8%; accordingly, the Ce^4+^/Ce^3+^ area ratio decreased from 1.56 to 1.34 (Figure S4A). These changes provide direct spectroscopic evidence consistent with the partial reduction of Ce^4+^ to Ce^3+^ by AA. Ce^3+^ is an excellent sensitizer for Tb^3+^, and the energy transfer process has been demonstrated in different host materials through excitation/emission spectra and lifetime analysis [39,40,41,42]. Time-resolved luminescence measurements showed that the lifetime of the Ce^3+^-related emission monitored at 350 nm decreased slightly from 9.167 to 8.750 μs after AA addition (Figure S4B), whereas the Tb^3+^ emission lifetime at 545 nm increased from 329.5 to 485.0 μs (Figure 3E). The complementary shortening of the Ce^3+^-related donor lifetime and lengthening of the Tb^3+^ acceptor lifetime provide kinetic evidence consistent with enhanced energy transfer from Ce^3+^ to Tb^3+^. Correspondingly, AA treatment markedly enhanced the characteristic Tb^3+^ emission at 545 nm (Figure 3D). Subsequently, to optimize the analytical performance of the probe, the fluorescence response to AA was evaluated using CeMOF@Tb probes prepared using different nominal Ce^4+^/Ce^3+^ precursor molar ratios. The results indicated that the probe prepared using a nominal Ce^4+^/Ce^3+^ precursor molar ratio of 6:4 showed the most remarkable enhancement in Tb^3+^ characteristic emission after the introduction of AA (Figure 3F and Figure S3). Accordingly, the CeMOF@Tb probe prepared using this nominal precursor ratio was used in the following experiments.

### 3.3. Sensing Performance Analysis for AA

Prior to evaluating the analytical performance, the effects of solution pH and reaction time were investigated. As shown in Figure S5A, the addition of AA produced the greatest enhancement in the Tb^3+^ emission at 545 nm when the solution pH was 6.0. This pH dependence may be partly associated with changes in the protonation state of AA, which varies with solution pH [43]. Therefore, pH 6.0 was selected for subsequent experiments. Under this condition, the fluorescence enhancement at 545 nm increased rapidly after the addition of AA and reached a relatively stable plateau at approximately 120 s (Figure S5B). Accordingly, an incubation time of 120 s was used for subsequent fluorescence measurements.

**Figure 4:**
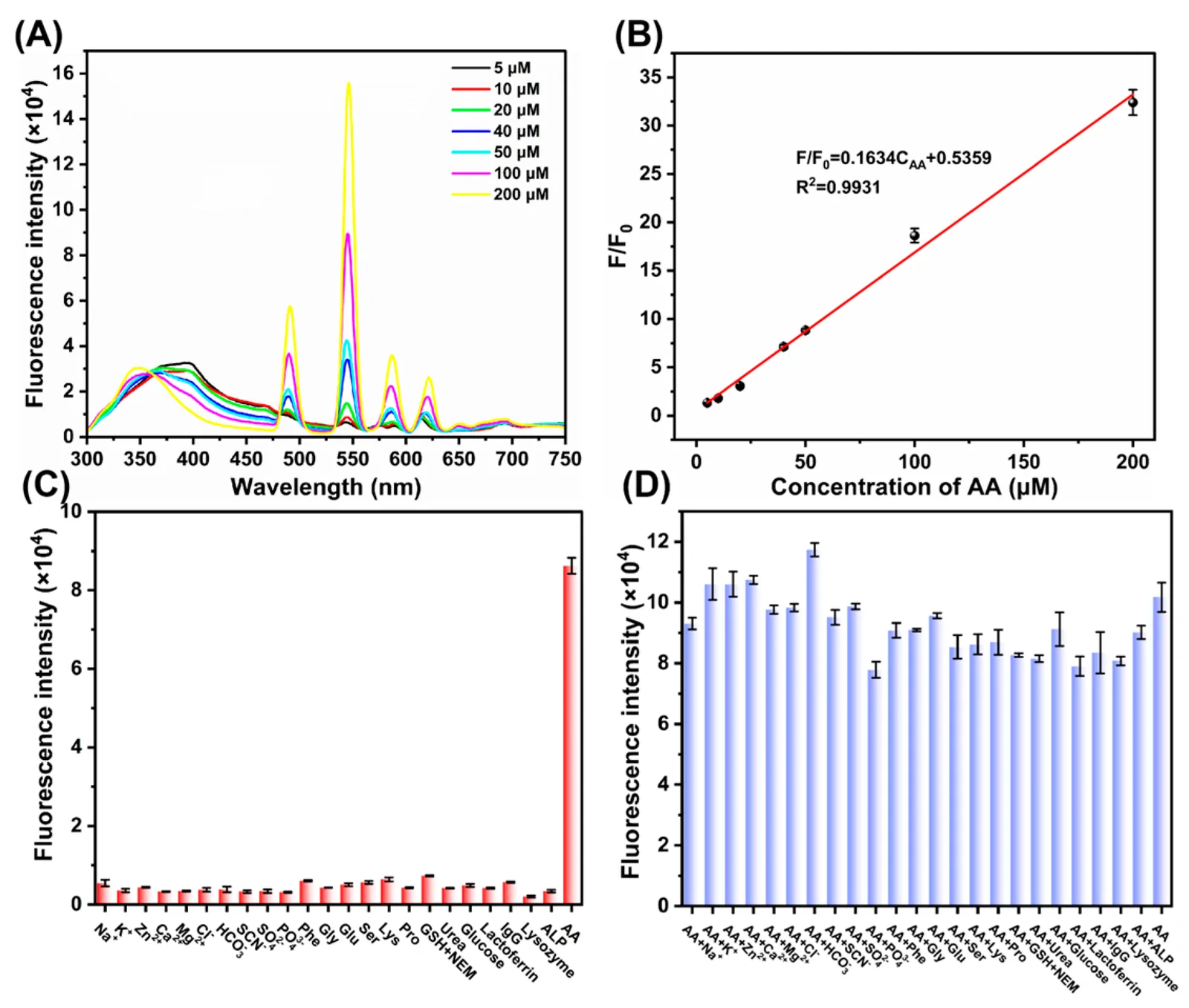
(**A**) Fluorescence spectra of CeMOF@Tb probe with different concentrations of AA. (**B**) Linear relationship between F/F_0_ and AA concentration. (**C**) Selectivity of the CeMOF@Tb probe toward AA against potential interferents. (**D**) Anti-interference performance of the CeMOF@Tb probe for AA detection in the presence of coexisting interferents.

Under the optimized conditions, CeMOF@Tb was used for the fluorescence detection of AA based on the redox-regulated energy transfer process. The Tb^3+^ emission at 545 nm gradually increased with increasing AA concentration (Figure 4A). A good linear relationship was obtained between the fluorescence response (F/F_0_) and AA concentration over the range of 5–200 μM, with the calibration equation F/F_0_ = 0.1634 C_AA_ + 0.5359 (R^2^ = 0.9931), where F and F_0_ represent the fluorescence intensities at 545 nm in the presence and absence of AA, respectively (Figure 4B). The limit of detection was calculated to be 0.7 μM according to the 3σ/k criterion (σ, the standard deviation of 11 blank samples; k, slope of the calibration curve). As summarized in Table S1, the linear range and LOD of the CeMOF@Tb sensing platform are comparable to those of several previously reported methods for AA detection. The selectivity of the CeMOF@Tb probe was evaluated against common reducing substances, ions, amino acids, sugars, and other components that may be present in biological samples. The limited response observed for GSH was associated with the use of NEM as a thiol-blocking reagent under the present assay conditions. As shown in Figure 4C, AA produced a pronounced increase in Tb^3+^ emission, whereas the tested potential interferents caused only minor fluorescence variations. Moreover, the response to AA was largely retained in the presence of these coexisting species, indicating good resistance to the potential interferents (Figure 4D). Different antioxidants may produce different fluorescence responses at the same molar concentration because of differences in their redox behavior and reaction rates. Accordingly, AA was used as the calibration standard, and the results were expressed as AA-equivalent TAC. The measured values therefore reflect the overall reducing capacity of the sample under the specified experimental conditions rather than the concentration of individual antioxidant species. These results support the application of CeMOF@Tb to the determination of AA-equivalent TAC in biological samples.

### 3.4. Determination of AA-Equivalent Antioxidant Capacity in Human Serum Samples

**Figure 5:**
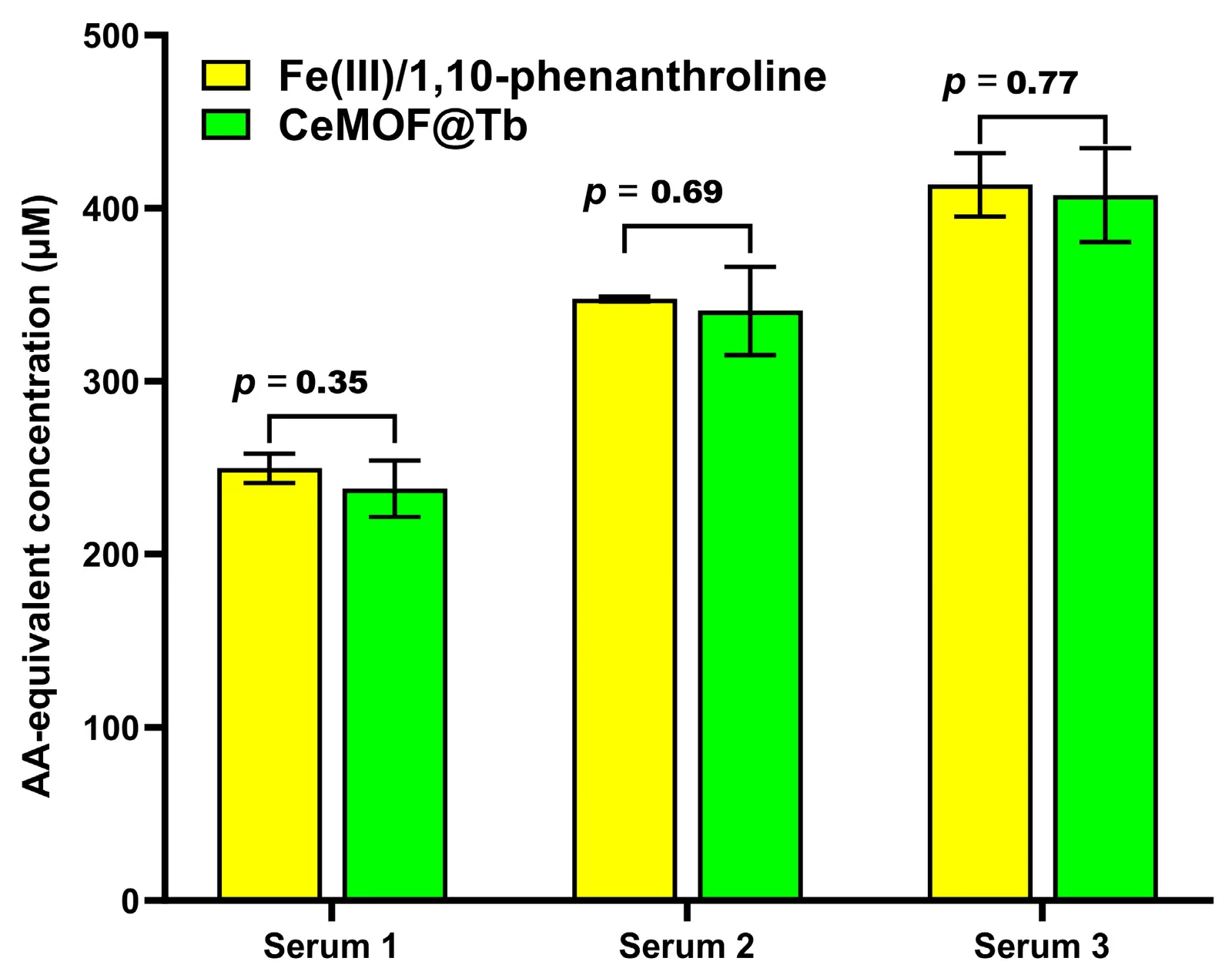
Comparison of AA-equivalent concentrations in three unspiked human serum samples determined using the proposed CeMOF@Tb fluorescence method and the Fe^3+^/1,10-phenanthroline UV-vis reference method. Data are presented as mean ± SD (n = 3). *p*-Values were calculated using separate two-sided unpaired Welch’s *t*-tests.

Unspiked serum samples were first analyzed directly using the CeMOF@Tb probe. Because 50 μL of serum was introduced into the final 250 μL reaction mixture, the values reported in Table 1 represent the AA-equivalent TAC after fivefold dilution. The measured values were 47.6 ± 3.3, 68.1 ± 5.1, and 81.5 ± 5.4 μM for Serum 1, Serum 2, and Serum 3, respectively. Standard addition experiments at final AA addition levels of 20 and 50 μM yielded recoveries of 91.6–122.6%, with RSD values ranging from 1.5% to 7.5% (Table 1). The corresponding values corrected to the original serum concentrations and the comparison with the Fe^3+^/1,10-phenanthroline method are presented in Table S2 and Figure 5.

Recovery (%) = [(Found value-unspiked value)/spiked value] × 100%

For independent validation, the same unspiked serum samples were analyzed using the Fe^3+^/1,10-phenanthroline UV-vis reference method described in Text S1 of the Supporting Information. The reference assay showed a linear response over the final assay concentration range of 10–125 μM (A_510_ = 0.01865 C_AA_ + 0.04087, R^2^ = 0.9995), as shown in Figure S6. As summarized in Table S2 and Figure 5, no statistically significant differences were observed between the two methods for the three serum samples (*p* = 0.35, 0.69, and 0.77).

## 4. Conclusions

In summary, a redox-responsive lanthanide metal–organic framework (CeMOF@Tb) probe was developed for the fluorescence determination of TAC, which integrates the Ce^4+^/Ce^3+^ redox couple as the recognition unit and Tb^3+^ as the luminescent center. CeMOF was prepared through mild aqueous coordination assembly, followed by post-synthetic incorporation of Tb^3+^. In the presence of antioxidants, Ce^4+^ is reduced to Ce^3+^, which weakens Ce^4+^-associated luminescence quenching and promotes energy transfer from Ce^3+^ to Tb^3+^. The redox-induced response leads to a marked enhancement in the characteristic Tb^3+^ emission at 545 nm, allowing the overall reducing capacity to be quantified. Under the optimized conditions, the proposed method exhibited an experimentally validated linear range of 5–200 μM and a statistically estimated LOD of 0.7 μM, together with good resistance to common coexisting interferents. The feasibility of the method for serum analysis was further supported by standard-addition recovery experiments and comparison with the UV-vis reference method. The study demonstrates that coupling a redox-active metal center with a lanthanide emitter provides an effective strategy for constructing responsive luminescent MOF sensors. The proposed platform offers a valuable approach for TAC analysis and may promote further applications in biological, food, and pharmaceutical antioxidant assessment.

## Figures and Tables

**Table 1:** Determination of AA-equivalent antioxidant capacity in human serum using the CeMOF@Tb fluorescence method.

Human Serum	AA Spiked (μM)	AA Found (μM)	RSD (%)	Recovery (%)
1	0	47.6 ± 3.3	6.9	-
	20	68.1 ± 4.1	6.0	102.7
	50	108.9 ± 7.3	6.7	122.6
2	0	68.1 ± 5.1	7.5	-
	20	86.4 ± 2.4	2.7	91.6
	50	122.6 ± 1.9	1.5	109.1
3	0	81.5 ± 5.4	6.6	-
	20	104.5 ± 3.1	3.0	115.2
	50	137.7 ± 3.6	2.6	112.5

Note: Values reported at an AA addition level of 0 μM represent the direct measurement of endogenous antioxidant capacity in the unspiked serum samples, expressed as AA-equivalent concentrations. Data are presented as mean ± standard deviation (SD) (n = 3).

## Data Availability

The data presented in this study are available on reasonable request from the corresponding author.

## Supplementary Materials

The following supporting information can be downloaded at: https://www.mdpi.com/article/10.3390/s26185711/s1, Text S1: Fe^3+^/1,10-phenanthroline UV-vis reference assay; Table S1: Comparison of different assays for detecting AA [44,45,46,47,48,49,50,51]; Table S2: Comparison of AA-equivalent concentrations in unspiked human serum samples determined using the UV-vis reference method and the proposed CeMOF@Tb fluorescence method; Figure S1: SEM images of CeMOF with different molar ratios between Ce^4+^ and Ce^3+^ (A, 10:0; B, 8:2; C, 6:4; D, 5:5; E, 4:6; F, 2:8); Figure S2: Powder X-ray diffraction patterns of CeMOF and CeMOF@Tb (n_Ce_^4+^/n_Ce_^3+^ = 6:4); Figure S3: Fluorescence responses of CeMOF@Tb with differentCe^4+^/Ce^3+^ molar ratios to AA; Figure S4: (A) High-resolution Ce 3d XPS spectrum and peak deconvolution of CeMOF@Tb after AA treatment. The relative Ce^3+^ and Ce^4+^ fractions were calculated from the integrated areas of their corresponding fitted components. (B) Lifetime decay curves of Ce^3+^ emission of the CeMOF@Tb probe at 350 nm before (black dots) and after (red dots) AA addition; Figure S5: (A) Effect of pH on the AA-induced fluorescence enhancement of CeMOF@Tb (ΔF_545_ = F_545_, AA − F_545_). (B) Time-dependent fluorescence intensity at 545 nm after the addition of AA at pH 6.0; Figure S6: Validation of the Fe^3+^/1,10-phenanthroline UV-vis reference method. (A) Absorption spectra obtained for AA standards over the concentration range of 10–125 μM. (B) Calibration curve between the absorbance at 510 nm (A_510_) and AA concentration. The calibration equation was A_510_ = 0.01865C_AA_ + 0.04087 (R^2^ = 0.9995). Calibration points are presented as mean ± SD from triplicate determinations (n = 3).
