# Supplementary material for: A Highly Sensitive Fluorescent Sensing Platform Utilizing Lanthanide Metal–Organic Frameworks for Total Antioxidant Detection
Source: Sensors (Basel). 2026 Sep 9;26(18):5711. doi: 10.3390/s26185711 (PMC13611057; doi:10.3390/s26185711)
Supplement: Supplementary file 1 [file sensors-26-05711-s001.zip › sensors-4505718-supplementary.pdf]

Supporting Information

A Highly Sensitive Fluorescent Sensing Platform Utilizing Lanthanide Metal–Organic Frameworks for Total Antioxidant Detection

Wanyang Zhou[a,1], Yuanqiao He[a,1], Shangqing Zhang[a], Yafei Chen[a], Haiyan Li[b]*, Mingli Chen[a]*

a Department of Chemistry, College of Sciences, Northeastern University, Shenyang, 110819, China

b College of Chemistry and Molecular Engineering, Northeast Agricultural University, Harbin, 150030, China

***Corresponding authors.**

Dr. Haiyan Li

E-mail: haiyanli175@163.com

Address: College of Chemistry and Molecular Engineering, Northeast Agricultural University, Harbin, 150030, China

Dr. Mingli Chen

E-mail: chenml@mail.neu.edu.cn

Address: Department of Chemistry, College of Sciences, Northeastern University, Shenyang, 110819, China

1 These authors contributed equally to this work

Text S1. Fe^{3+}/1,10-phenanthroline UV-vis reference assay

AA standard solutions were freshly prepared before use. A 2.5 mM $FeCl_3 \cdot 6H_2O$ working solution was prepared in 0.01 M HCl. A 5.0 mM 1,10-phenanthroline monohydrate working solution was prepared in ultrapure water. A 10 mM HEPES solution adjusted to pH 4.0 was prepared separately.

For each measurement, 50 μL of AA standard solution was mixed with 50 μL of the $FeCl_3$ working solution, followed by the addition of 50 μL of the 1,10-phenanthroline working solution and 100 μL of the 10 mM HEPES solution adjusted to pH 4.0. The final reaction volume was 250 μL. Under these conditions, Fe^{3+} was maintained in molar excess over AA throughout the investigated concentration range. After thorough mixing, the UV-vis absorption spectrum was recorded over 450–600 nm using a UV-3600i Plus spectrophotometer (Shimadzu Corporation, Kyoto, Japan) equipped with a quartz microcuvette with a 1.0 cm optical path length. The absorbance at 510 nm (A_{510}) was used as the analytical signal.

AA standards with final assay concentrations of 10, 20, 40, 50, 75, 100, and 125 μM were used to construct the calibration curve. The reagent blank was prepared under identical conditions, except that the AA standard solution was replaced with 50 μL of ultrapure water. For serum analysis, 50 μL of the unspiked serum sample was used in place of the AA standard solution and treated under the same conditions. The AA-equivalent concentrations used for method comparison were multiplied by a dilution factor of 5 and expressed on an original-serum basis. All measurements were performed in triplicate.

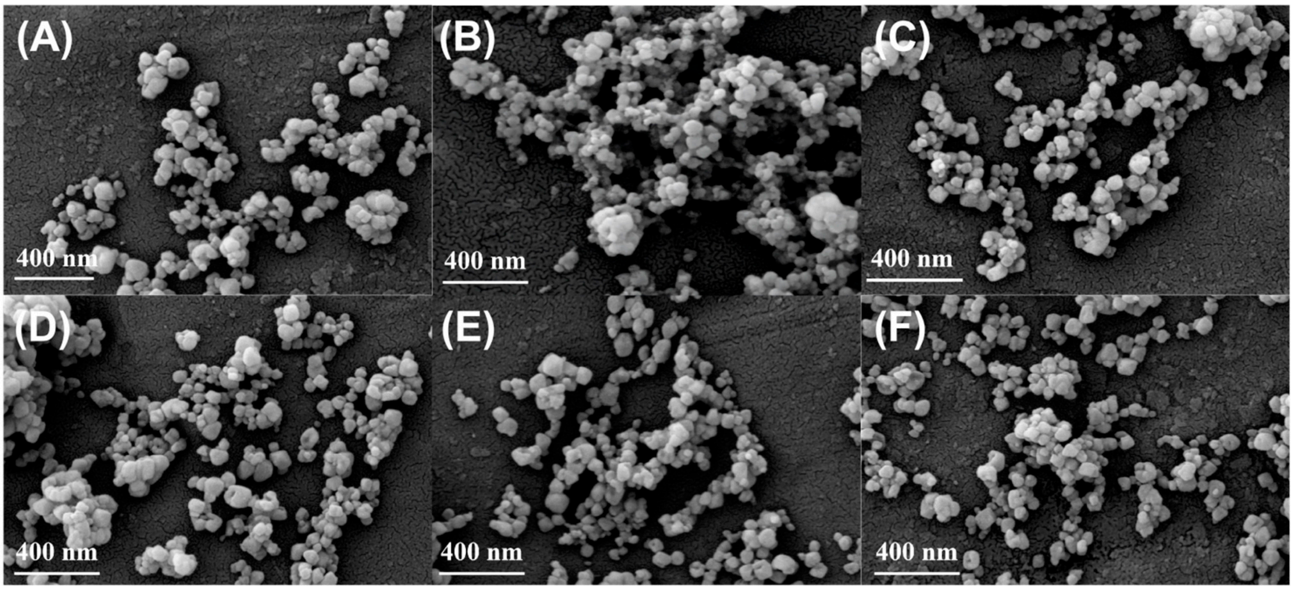

Figure S1. SEM images of CeMOF with different molar ratios between Ce^{4+} and Ce^{3+} (A, 10:0; B, 8:2; C, 6:4; D, 5:5; E, 4:6; F, 2:8).

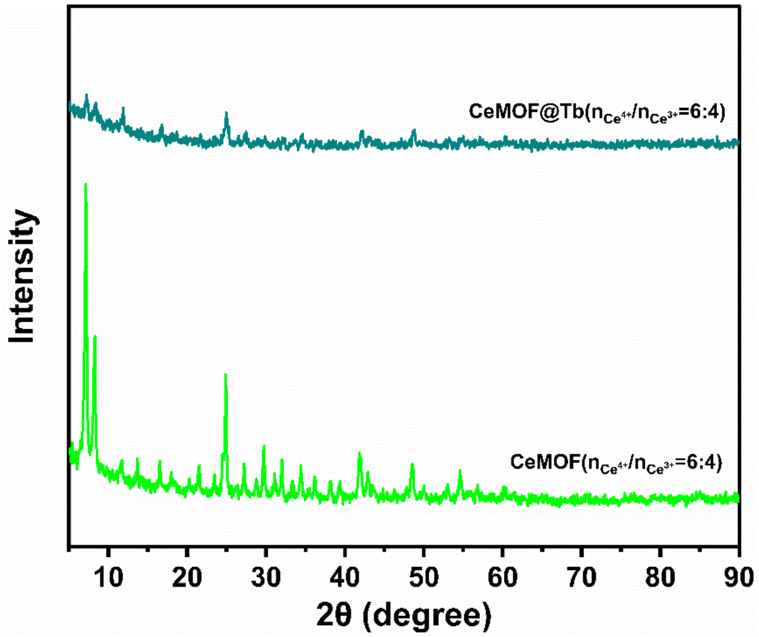

Figure S2. Powder X-ray diffraction patterns of CeMOF and CeMOF@Tb (n_{Ce}^{4+}/n_{Ce}^{3+}=6:4).

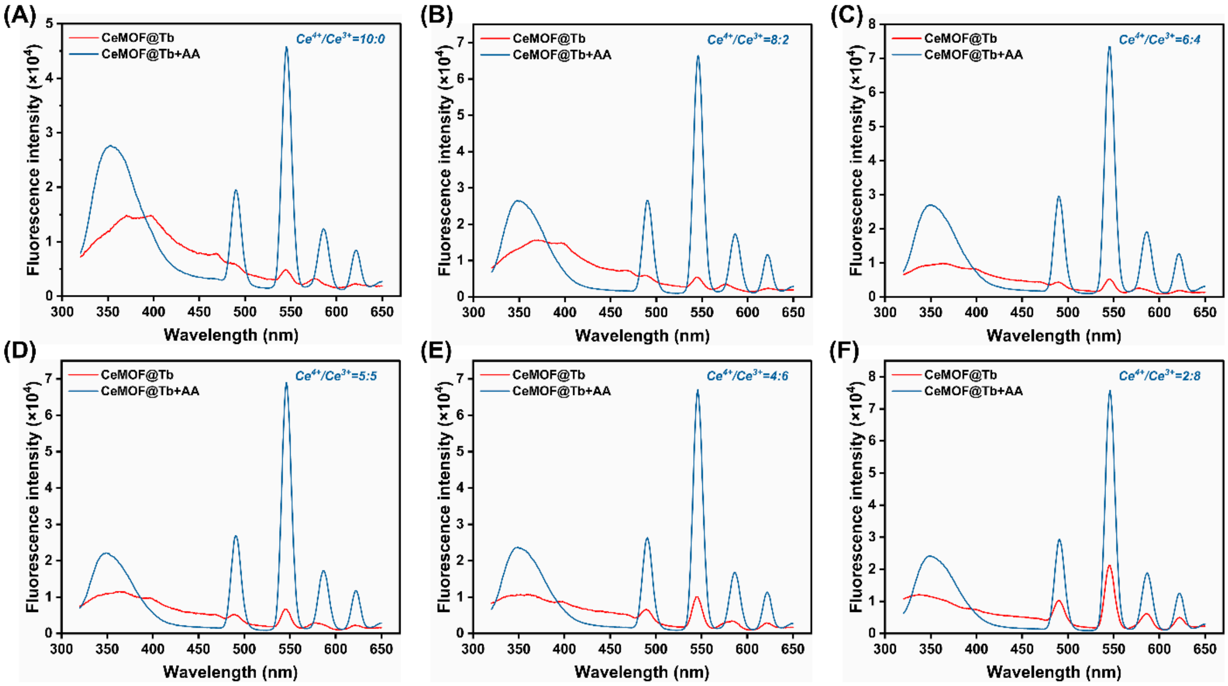

Figure S3. Fluorescence response of CeMOF@Tb with different Ce^{4+}/Ce^{3+} molar ratios to AA.

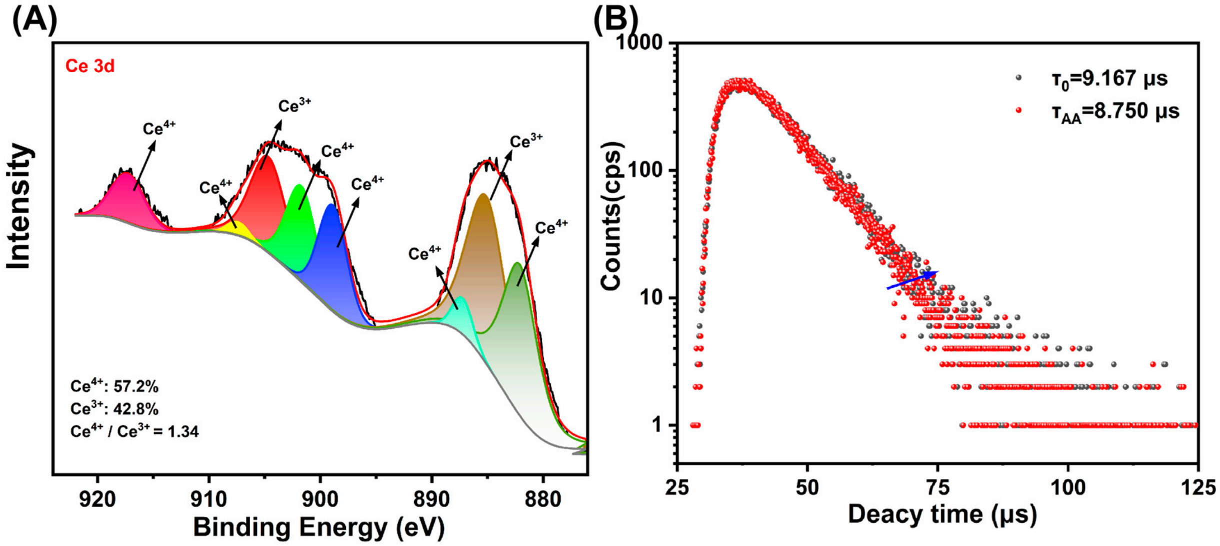

Figure S4. (A) High-resolution Ce 3d XPS spectrum and peak deconvolution of CeMOF@Tb after AA treatment. The relative Ce^{3+} and Ce^{4+} fractions were calculated from the integrated areas of their corresponding fitted components. (B) Lifetime decay curves of Ce^{3+} emission of the CeMOF@Tb probe at 350 nm before (black dots) and after (red dots) AA addition.

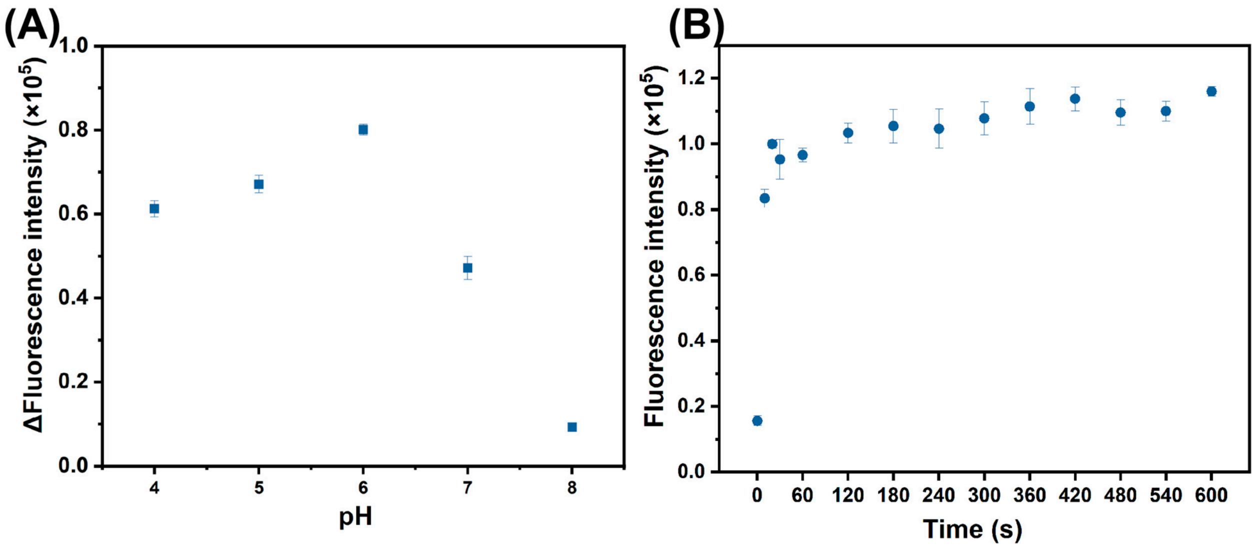

Figure S5. (A) Effect of pH on the AA-induced fluorescence enhancement of CeMOF@Tb (ΔF_{545} = $F_{545,AA} - F_{545}$). (B) Time-dependent fluorescence intensity at 545 nm after the addition of AA at pH 6.0.

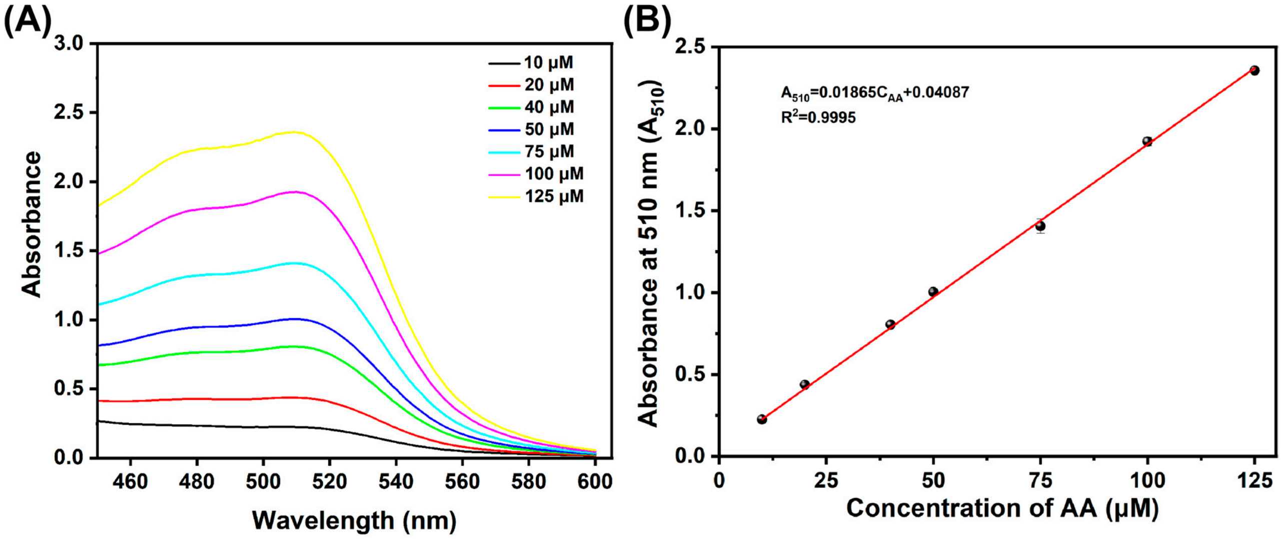

Figure S6. Validation of the Fe^{3+}/1,10-phenanthroline UV-vis reference method. (A) Absorption spectra obtained for AA standards over the concentration range of 10–125 μM. (B) Calibration curve between the absorbance at 510 nm (A_{510}) and AA concentration. The calibration equation was A_{510} = 0.01865C_{AA} + 0.04087 (R^2 = 0.9995). Calibration points are presented as mean ± SD from triplicate determinations (n = 3).

Table S1. Comparison of different assays for detecting AA.

Materials	Detection Mode	Linear Range (μM)	Detection Limit (μM)	Ref.
$(HDMEA)_9Na_{18}H_3$ $[Bi_{10}Eu_2(H_2O)_4(DMEA)_2(H_2OA)_4W_{10}O_{36}][B\text{-}\alpha\text{-}SbW_9O_{33}]_6\cdot 124H_2O$	Fluorescence	40–200	0.038	[1]
ZIF-8/Ru@TPE	Fluorescence	5–100	2.71	[2]
Fe-CDs	Fluorescence	5–100	1.8	[3]
Fe-CDs	Colorimetry	2.5–60	0.9	[3]
FeCo-CDs	Fluorescence	25–150 150–300	0.35	[4]
FeCo-CDs	Colorimetry	5–40	0.39	[4]
PCN-224@PAA-Ce	Fluorescence	0.5–50 50–100	0.73	[5]
PCN-224@PAA-Ce	Photothermal	3–80	1.4	[5]
Co-N-C	Electrochemical	100–2000	4.83	[6]
CFE/CNT-COOH/PFC-71	Electrochemical	100–600	16.8	[7]
AuNPs@2-MBQ@COFs	SERS	1–10000	0.66	[8]
CeMOF@Tb	Fluorescence	5–200	0.7	This work

Table S2. Comparison of AA-equivalent concentrations in unspiked human serum samples determined using the UV-vis reference method and the proposed CeMOF@Tb fluorescence method.

Human Serum	Fe^{3+}/1,10-Phenanthroline Method (μM)	CeMOF@Tb Method (μM)	*p*-Value
1	249.7±8.4	237.9±16.3	0.35
2	347.2±1.4	340.5±25.6	0.69
3	413.5±18.3	407.5±27.0	0.77

Note: The AA-equivalent concentrations were corrected for the fivefold dilution of serum in the final 250 μL assay mixtures and are reported on an original-serum basis. Results are presented as mean ± SD from triplicate determinations (n = 3). *p*-Values were calculated using separate two-sided unpaired Welch's t-tests. No statistically significant differences were observed between the two methods for any of the three serum samples (all $p > 0.05$).

References

1. Zhang, Y.; Yang, S.; Pu, Y.; Chen, L.; Zhao, J., Heterometallic BiIII-EuIII Cluster-embedded Antimonotungstate: Rational Design and Dual-Mode Ascorbic Acid Detection Integrating Fluorescent Sensing and Smartphone-Assisted Onsite Assay. *ACS Applied Materials & Interfaces* **2026,** ***18*****, 411**96-41208
2. Wang, X.; Li, S.; Zhang, S.; Sun, Q.; Cheng, Q.; Bao, K.; Xia, B.; Wei, H., AIE Fluorescent Nanozyme-Enabled Ratiometric Sensing Platform for Selective Detection of Ascorbic Acid and Alkaline Phosphatase. *Analytical Chemistry* **2026,** *98*, 5623-5633.
3. Li, Y.; Cao, F.; Deng, X.; Li, J.; Wang, Z.; Yu, X., Simultaneous Total Antioxidant Capacity Assessment and Ascorbic Acid Accurate Quantification Utilizing a H_2O_2-Independent Colorimetric-Fluorescent Sensor Array. *Food Chemistry* **2026,** *499*, 147257.
4. Wu, S.; Yu, Z.; Zhao, J.; Wang, Y.; Nan, J.; Xu, L.; Li, X.; Yang, L.; Dong, S., Fluorescent Nanozyme Based on Bimetallic Fe/Co-Doped Carbon Dots for Dual-Mode Detection of Ascorbic Acid and Hydrogen Peroxide. *Analytical Chemistry* **2025,** *97*, 15320-15328.
5. Li, M.; Zhang, S.; Li, H.; Chen, M., Cerium/Polyacrylic Acid Modified Porphyrin Metal-Organic Framework as Fluorescence and Photothermal Sensor for Ascorbic Acid Measurement. *Talanta* **2023,** *252*, 123825.
6. Jin, J.; Wei, J.-J.; Gu, Z.-Y.; Zhang, Y.-X.; Lu, Z.-Y.; Zhang, Q.-W.; Wan, J.-J.; Shi, G.-Y.; Xia, X.-H.; Shi, Y., Dual-Mode Sensor Based on a Single-Atom Cobalt Catalyst for Simultaneous Electrochemical and Colorimetric Detection of Bioactive Small Molecules. *Analytical Chemistry* **2025,** *97*, 8617-8624.
7. Zheng, Z.; Liu, N.; Lu, J.; Zhou, X.; Song, Z.; An, Y.; Lu, L.; Zhao, P.; Tao, J., Hydrogen-Bonded Organic Framework Enhanced Antifouling Property for Efficient In Situ Electrochemical Assay of Cerebral Ascorbic Acid. *Small* **2024,** *20*, 2407101.
8. Chen, Z.-C.; Xu, H.-B.; Chen, H.-Y.; Zhu, S.-C.; Huang, W.-F.; He, Y.; Hafez, M. E.; Qian, R.-C.; Li, D.-W., AuNPs-COFs Core-Shell Reversible SERS Nanosensor for Monitoring Intracellular Redox Dynamics. *Analytical Chemistry* **2022,** *94*, 14280-14289.
